# Asymmetry and integration of cellular morphology in *Micrasterias compereana*

**DOI:** 10.1186/s12862-016-0855-1

**Published:** 2017-01-03

**Authors:** Jiří Neustupa

**Affiliations:** Department of Botany, Faculty of Science, Charles University, Prague, Czech Republic

**Keywords:** *Desmidiales*, Geometric morphometrics, Green algae, *Micrasterias*, Morphological asymmetry, Morphological integration

## Abstract

**Background:**

Unicellular green algae of the genus *Micrasterias* (Desmidiales) have complex cells with multiple lobes and indentations, and therefore, they are considered model organisms for research on plant cell morphogenesis and variation. *Micrasterias* cells have a typical biradial symmetric arrangement and multiple terminal lobules. They are composed of two semicells that can be further differentiated into three structural components: the polar lobe and two lateral lobes. Experimental studies suggested that these cellular parts have specific evolutionary patterns and develop independently. In this study, different geometric morphometric methods were used to address whether the semicells of *Micrasterias compereana* are truly not integrated with regard to the covariation of their shape data. In addition, morphological integration within the semicells was studied to ascertain whether individual lobes constitute distinct units that may be considered as separate modules. In parallel, I sought to determine whether the main components of morphological asymmetry could highlight underlying cytomorphogenetic processes that could indicate preferred directions of variation, canalizing evolutionary changes in cellular morphology.

**Results:**

Differentiation between opposite semicells constituted the most prominent subset of cellular asymmetry. The second important asymmetric pattern, recovered by the Procrustes ANOVA models, described differentiation between the adjacent lobules within the quadrants. Other asymmetric components proved to be relatively unimportant. Opposite semicells were shown to be completely independent of each other on the basis of the partial least squares analysis analyses. In addition, polar lobes were weakly integrated with adjacent lateral lobes. Conversely, higher covariance levels between the two lateral lobes of the same semicell indicated mutual interconnection and significant integration between these parts.

**Conclusions:**

*Micrasterias* cells are composed of several successively disintegrated parts. These integration patterns concurred with presumed scenarios of morphological evolution within the lineage. In addition, asymmetric differentiation in the shape of the lobules involves two major patterns: asymmetry across the isthmus axis and among the adjacent lobules. Notably, asymmetry among the adjacent lobules may be related to evolutionary differentiation among species, but it may also point out developmental instability related to environmental factors.

**Electronic supplementary material:**

The online version of this article (doi:10.1186/s12862-016-0855-1) contains supplementary material, which is available to authorized users.

## Background

In organisms with modular body plans, composed of multiple repeated parts, the concepts of morphological symmetry and integration are inherently related. Typical organisms with this architecture are vascular plants with multiple repeated organs symmetric to each other [1, 2]. Such symmetric morphological units have joint developmental origins at the molecular level and they can be viewed as repetitions of the same motif.

Morphological integration has been defined as the cohesion among traits that results from interactions of morphological processes and structures [3]. In parallel, modularity is based on the quantification of the differences in integration of different structural components. The repeated units, jointly forming a single biological structure, may have widely different levels of mutual morphological integration. Especially parts that develop diachronically, e.g. in different life-cycle phases or ecological conditions, may profoundly differ in their morphological integration levels. Such patterns may constitute key constraints in the evolution of biological forms, because they facilitate evolutionary change in only a part of a structure, thus leading to asymmetric differentiation of shape features. Evolvability of organism morphology is then constrained both by translational symmetry, i.e. multiple repetitions of symmetric parts based on joint developmental networks at the molecular level, and by different integration levels among the different regions.

Most of the actual data on phenotypic variation of multiple symmetric modular parts have been acquired through studies on multicellular organisms, such as vascular plants [4, 5] and segmented [6] or colonial invertebrates [7]. In multicellular organisms, morphogenesis is primarily related to patterns of interaction among the cells, the control of cellular differentiation, adhesion, and tissue growth [3]. Variation in these processes then leads to different patterns of morphological symmetry and asymmetry in macroscopic structures such as leaves [8] or body segments [6], which constitute the developmental modules of the organism. Notably, asymmetric morphological variation of these structures can be partitioned into directional asymmetry, i.e. mean asymmetric deviation from a perfectly symmetric shape, and fluctuating asymmetry, which represents random individual variation around the asymmetric mean. Asymmetric morphological variation has therefore also been considered as a measure of developmental instability [1, 9].

There is considerably less data on both the integration and symmetric shape variation of unicellular morphologies. In their pioneering study, Medarde and her colleagues identified three morphological modules comprising the head of the sperm cells of mice [10]. Interestingly, the modules corresponded to cytoskeleton differentiation beneath the plasma membrane of the cell, and the authors concluded that the structural heterogeneity of the cytoskeletal mesh was directly related to the morphological integration patterns of the sperm cell shape. However, despite its modular arrangement, the mammalian sperm cell is a compact morphological structure, i.e. the morphogenesis of its cellular parts is synchronic and the regions, which correspond to structural modules, are spatially tightly related. Conversely, several protist lineages possess vegetative cells with a complicated multi-level symmetric morphology, possibly related to the different levels or patterns of integration among individual cellular parts. The desmids (Desmidiales, Zygnematophyceae) have been established as a model group for investigation of morphological symmetry at the cellular level [11, 12]. They have also been a prime model system for the study of the intracellular mechanisms of plant cell morphogenesis [13–16] and mathematical modelling of the cellular growth and development [17–19]. The cellular shapes of many desmids typically have extremely low isoperimetric quotients, i.e. their cellular outline deviates strongly from circularity [20–22]. The shapes of the mature cells are rigid, due to their cellulose secondary cell wall. Desmid cells are typically composed of two symmetric halves or semicells, joined by a narrow central tunnel or isthmus, which contains the interphase nucleus. Desmids also have a peculiar asexual reproduction process. Cellular division occurs in the isthmus region and separated semicells develop their ‘daughter’ semicell counterparts [21]. Consequently, each cell within a population is composed of two halves, symmetric to each other but different in age. In addition, the individual semicells of many desmid lineages typically have a bilateral symmetric morphological arrangement. Most members of the monophyletic *Micrasterias* lineage [23] possess flat semicells with numerous bilaterally symmetric lobes and lobules. *Micrasterias* cells are composed of four symmetric quadrants, an arrangement known as disymmetry or biradial symmetry [24]. While the two quadrants that form a single semicell develop simultaneously, the morphogenesis of the opposite quadrants may have occurred several generations earlier. Most of the asymmetric morphological variation within *Micrasterias* cells can be ascribed to differences between semicells, with the adjacent quadrants of a single semicell identical in shape, but different from the quadrants of an opposite semicell [11, 12]. This dominant pattern of the *Micrasterias* cell shape asymmetry has been explained either by the direct effects of external factors such as temperature [25–27], or by an allometric effect based on the size differences among the semicells of a single species [22].

**Fig. 1:**
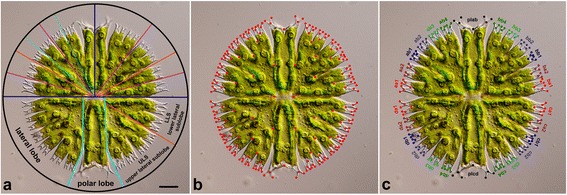
**a** A scheme showing lobes and lobules of *Micrasterias compereana*. The differentiation of the lower semicell into the polar lobe, two lateral lobes, and multiple sublobes is illustrated. The upper semicell shows differentiation of the lateral lobes into lobules of the 3^rd^ and 4^th^ order. **b** Position of 208 landmarks on the cell of *Micrasterias compereana*. **c** Position of landmarks on terminal lobules designated as units for the analyses of matching symmetry and morphological integration. Scale bar = 25 μm

However, a purely geometric description of the *Micrasterias* morphology as a disymmetric structure with two-fold object symmetry does not fully correspond to the actual morphogenetic pattern of developing semicells. Waris and Kallio [28] showed that each semicell probably comprises three main developmental components: two lateral lobes that are bilaterally symmetric to each other, and one polar lobe (Fig. 1a). They also illustrated that the presence of the polar lobe was essential for the morphogenesis of developing semicells. Conversely, development of the lateral lobes could be experimentally blocked without any immediate effect on cell viability. In many cases, such teratogenic semicells then produced their own ‘mirror images’, which resulted in clonal populations of uniradiate cells, lacking a single lateral lobe, or aradiate clones possessing only the polar lobe [29, 30]. Waris and Kallio therefore suggested that the number of lobes was primarily controlled by the cytoplasmic inheritance between the older and newly developing semicells. However, this cytoplasmic inheritance of the teratogenic morphology is probably limited to several asexual generations, because Gärtner and Meindl [31] showed that the uniradiate *Micrasterias thomasiana* population gradually reverted to its wild-type biradiate morphology after a series of mitotic cell divisions. Interestingly, Kallio and Lehtonen [30] also showed that when cells were enucleated with UV radiation just prior to the vegetative division, the resulting semicells developed at least three rudimental lobes that represented the basis of the polar lobe and two lateral lobes. However, development of the enucleated cells did not continue any further and thus the species-specific morphology of the terminal lobules could not be achieved.

It has been unclear whether morphology of the non-teratogenic semicells, i.e. possessing both the polar lobe and two lateral lobes, could also be related to their older counterparts. This was previously proposed by several authors on the basis of microscopic observations of natural desmid populations [21, 32], but has not been experimentally confirmed. Such pattern would suggest that the ‘cytoplasmic inheritance’, observed in mutant *Micrasterias* cells at the level of all cellular lobes [28, 30], also affects morphogenesis of individual terminal lobules. In that case, it should be possible to find patterns of increased morphological symmetry between corresponding lobules of opposite semicells, and significant morphological integration.

Kiermayer [13, 33] demonstrated that an initial pattern of the three-lobed semicell shape is already present at an early stage of the cell division in the septum membrane separating the freshly divided semicells. The initial pattern was visualised under turgor reduction when the primary wall material is deposited at particular areas of the septum membrane, but cannot be properly incorporated into the developing primary cell wall. In these conditions, the wall material is deposited in a patterned way that is characterised by minimum zones corresponding to later incisions among the major semicell lobes [14]. Likewise, the areas corresponding to lobes were characterized by increased deposition of cell wall material. Thus, Kiermayer [33] assumed that the plasma membrane of the septum bears specific receptors that serve as recognition sites for the vesicular transport of cell wall material in actively growing areas of the developing semicells. This type of transport occurs via actin cytoskeleton microfilaments [34–36] rather than via microtubules [13, 14]. A recent study showed that vesicular transport during semicells morphogenesis is regulated by MdRABE1, a protein belonging to the Rab family, which may have a potential role in signalling processes associated with cell shape formation [37]. Pattern formation at actively growing areas is also accompanied by a local influx of Ca^2+^ ions [14, 16, 38]. During morphogenesis, this calcium influx tightly reflects the branching pattern of the developing lobes and lobules [16]. Although the exact role of Ca^2+^ in the morphogenetic process has not been fully explained, it is assumed to be important for fusion of the secretory vesicles at the actively growing sites, as well as for regulating flexibility of the primary cell wall. The cell wall is able to bind relatively high amounts of calcium during its development, which eventually leads to its stiffening and the termination of local cellular growth [16].

Using laser treatment on different membrane areas of developing semicells, Lacalli [39, 40] showed that the plasma membrane includes specific microsites of key importance for morphogenesis of individual lobes or lobules. These studies showed that laser treatment at the early stages of lateral lobe development halted morphogenesis at a particular site, so that it was more likely to be repaired in the later stages, leading to a semicell with non-teratogenic morphology. Conversely, lasing the membrane in later developmental stages typically resulted in a breakdown of the morphogenesis of a particular terminal lobule. Interestingly, laser treatment of the central part of the developing semicell prevented formation of the polar lobe, but the lateral lobes remained unaffected. Thus, these experiments demonstrated that laser damage to developing *Micrasterias* semicells was distinctly localized to individual lobes, indicating that they develop relatively independent [19, 40]. In parallel, Harrison and co-workers suggested that the mechanistic basis of *Micrasterias* morphogenesis might be explained by differential growth of the plasma membrane, driven by two-morphogen reaction–diffusion activity, controlling both the tip growth of individual lobules, as well as their dichotomic branching [17, 18, 41]. Interestingly, compartmentalisation of the developing semicell into relatively independent lobes and lobules was an inherent aspect of their mathematical model. Thus, differential patterning of the cell surface in the later stages of the morphogenesis occurred in multiple actively developing centres with minimal mutual coordination [42]. Such a morphogenetic pattern corresponds well with the idea of several weakly integrated developmental modules within a single *Micrasterias* semicell.

Recent developments in the quantitative analysis of morphology make it possible to evaluate many of the phenomena observed in experimental studies, and to test hypotheses that explain the observed morphogenetic patterns. Decomposition of cell shape asymmetry based on the parallel analysis of all symmetry transformations of a single symmetry group by geometric morphometrics leads to the quantification of different patterns of asymmetric variation within studied populations [1, 24]. The components of the asymmetric variation occupy mutually orthogonal subspaces of the total morphospace of the studied dataset. Thus, these subspaces represent the unique contribution of a particular asymmetric pattern to the overall morphological variation [1]. There are two types of analyses for shape asymmetry. *Object symmetry* denotes the situation where the object itself is symmetric, such as the front view of the *Micrasterias* cell (Fig. 1), with axes of symmetry dividing it into several symmetric parts. The morphometric analysis can then quantify the morphological asymmetry attributed to both major axes of the biradial front views of cells, i.e. asymmetry across the isthmus plane between semicells and among adjacent quadrants within semicells. Finally, an additional subset is occupied by combination of these two axes, which yields the transversally asymmetric arrangement of the quadrants [11, 12].

Alternatively, *matching symmetry* describes a pattern with spatially separated symmetric copies of a single structure, such as the human hands or fly wings [1]. If the terminal cellular lobules of *Micrasterias* are considered as basic units and the analysis concerns their separate shapes (Fig. 1c), there may be several additional asymmetric components, defined by asymmetry among the lobules within the quadrants. The number of these intra-quadrant asymmetric components, as well as their combinations with the inter-quadrant components, depends on the particular species-specific morphology.

This partition of the asymmetric variation into separate components, both within and among the semicells, may be used for evaluation of the effects of individual morphogenetic processes on the total cellular asymmetry. For example, we may be able to evaluate whether morphology of the older semicell has any discernible effect on the morphology of its younger counterpart. In addition, an analysis at the level of the terminal lobules may be useful for the assessment of shape asymmetry within the lateral lobes and its comparison with other asymmetric subspaces of the overall morphospace. Published mathematical models of the *Micrasterias* morphogenesis generally describe symmetric branching of the growing lobules. Thus, such a pattern should correspond to the random distribution of the asymmetric deviations in lobule shape within a single cellular lobe. Pronounced intra-lobe asymmetry among the terminal lobules would indicate that branching processes during tip growth of the developing semicells may not lead to identical shapes of the terminal lobules, contradicting classical descriptions of cellular morphology [20, 43] and theoretical models [17, 18]. The analysis should illustrate which of the theoretically possible intracellular shape asymmetry patterns are preferred in actual cellular morphogenesis. These preferred directions of the variation in morphospace would then represent the substrate for microevolutionary processes. In other words, it may illustrate how the morphological complexity of cells is channelled towards asymmetric differentiation among individual parts, representing preferred directions in the evolution of the cellular shape as a whole.

In this study, which to our knowledge is the first of its kind, complex patterns of (a) symmetry among the terminal lobules of *Micrasterias* cells are investigated. These lobules may be reflected across multiple symmetry axes, constituting a unique model system for the investigation of their matching symmetry. They may be analysed in a joint Procrustes superimposition that includes all the symmetric copies from each of the specimens under study. The resulting matrix of the tangent Procrustes distances (PD), that is, differences in shape of the lobules, may then be partitioned according to the main axes of cellular symmetry. It should be mentioned that cellular symmetry, typical for desmids and many other microalgae and protists, differs from the symmetric arrangement of body parts of multicellular organisms in its inherent ambiguity with regard to front and back, up and down, and left and right [1, 11]. Thus, the morphometric analysis considers just the total asymmetry spanned by individual axes as the deviation from the total symmetry, but it cannot identify the components of directional and fluctuating asymmetry that are usually separated in studies of morphological asymmetry of higher plants or animals [5, 6, 44]. However, one advantage of working with a unicellular model is that a clonal population can easily be established and kept for many generations so that the genetic variation can be removed.

The aim of the study was to address the following questions relating to object and matching symmetry of the quadrants and terminal lobules of cells. What are the proportions of the total asymmetry among the quadrants and terminal lobules between the semicells; that is, between the diachronically developing cellular parts? Is there any detectable effect of the older semicell on its younger counterpart, leading to significant more similarity than between semicells belonging to different cells? Is there any significant part of the shape asymmetry that can be attributed to variation within the semicells? What part of this variation could be ascribed to asymmetry within the quadrants, i.e. to morphogenetic differentiation among the lobules forming a single lateral lobe?

In addition to partition of the asymmetric variation, the terminal lobules were also used as fundamental units for the analysis of their mutual covariation patterns. In other words, I wanted to ascertain how the different parts of the *Micrasterias* cell covary with regard to their morphological variation. Significant morphological integration between two parts may not necessarily be connected with their high level of symmetry. Two highly asymmetric structures obviously may or may not be significantly integrated in their morphological variation. However, any two ideally symmetric structures, i.e. those varying only among individuals but with identical shapes within specimens, would also be totally integrated. Matrices of signed fluctuating asymmetry in bilaterally symmetric structures of important animal models have often been used for quantification of the developmental integration [3, 45]. However, the cellular parts are typically unsigned and, thus, their asymmetry cannot be distinguished into directional and fluctuating components. Therefore, I used the total non-allometric shape variation of the cells for evaluation of their integration patterns. This approach, also referred to as static integration analysis, relies on comparison of individuals from a homogenous sample, i.e. from a single species and ontogenetic stage [45]. As a result, it should be possible to determine which cellular parts of the studied *Micrasterias* model population covary in coordinated fashion and which of them are mutually independent. In this regard, I asked whether there will be any significant covariation between lobules on two opposite semicells. Likewise, will there be any significant integration among the lobules developing synchronically, but in opposite lateral lobes of a single semicell? And finally, can the basic structuring of the *Micrasterias* semicell into the polar lobe and two lateral lobes, which are further divided into two lobules, be discerned in differing integration patterns among the terminal lobules? Answers to these questions would lead to a better understanding of the morphogenetic interactions that produce the complex cellular shapes of these microalgae. In this way, geometric morphometrics would complement the earlier experimental studies and mathematical modelling of the cellular morphogenesis in this fascinating unicellular organism. In addition, it would shed more light on morphogenetic patterns and constraints that underlie the morphological evolution of this microalgal lineage, which produced one of the most remarkable cellular shapes in the plant kingdom.

## Methods

### Cultivation and data acquisition

The studied dataset comprised 68 mature cells taken from CAUP K608, a clonal strain of *Micrasterias compereana*. This strain, which has been used as a holotype for the taxonomic description of the species by Neustupa et al. [46], was originally isolated in 2011 from oligotrophic peaty pools near Étang Hardy, Aquitaine, France (43°43′08.60″N, 01°22′09.42″W). It was cultivated in 250 ml Erlenmeyer flasks with approximately 125 ml of the MES-buffered DY IV liquid medium at 22 °C and illuminated at 40 μmol photons m^−2^ s^−1^ with 18 W cool fluorescent tubes (Philips TLD 18 W/33), at a light:dark (L:D) regime of 12:12 h.

The cells were photographed at 200× magnification on an Olympus BX51 light microscope with Olympus DP27 digital photographic equipment. In total, 208 structurally corresponding landmarks were depicted on the front-view images of the cells (Fig. 1b, Additional file 1) using TpsDig software, ver. 2.15 [47]. To assess the measurement error, all landmarks were digitised twice. In the first digitisation, the landmarks were registered clockwise starting from the left margin of the cellular isthmus. Conversely, the second digitisation proceeded counter clockwise from the same starting point and the landmarks were relabelled to match the labels of the first digitisation.

### Analysis of cellular symmetry

For object symmetry analysis, the landmark configurations were subjected to four symmetry transformations: (1) identity; reflections of the landmark configurations across the (2) vertical and (3) horizontal axes; and finally, (4) reflection across both these axes. In parallel, individual reflections were accompanied by appropriate re-labelling of the landmarks to ensure their consistent order. Consequently, each cell was represented by four configurations, differing by the mutual position of each of the quadrants. The resulting dataset consisted of 68 × 4 = 272 configurations. Individual patterns of object symmetry and asymmetry occupied the orthogonal subspaces of the overall shape space. In the case of *Micrasterias* cells, these four subspaces are as follows: (1) totally symmetric variation with all four quadrants varying in an identical fashion; (2) asymmetric variation across the isthmus axis, which differentiates between shape features of both semicells; (3) variation across the vertical axis differentiating the quadrants of semicells, while keeping the shape features of adjacent quadrants across the isthmus axis identical; and (4) asymmetry across both axes resulting in variation patterns that keeps the transversally positioned quadrants identical.

The generalised Procrustes analysis (GPA) was followed by principal component analysis of the Procrustes coordinates in the package *shapes* ver. 1.1-11 [48] in R ver. 3.2.3 [49]. Patterns of variation spanned by the two most important axes in each of the four subspaces were illustrated by thin-plate splines in TpsRelw ver. 1.49 [47]. Relative amounts of the four subspaces (symmetry and three asymmetric patterns) were quantified by summing up the variation spanning the principal components of each of these subsets. In addition, scores of individual cells on the principal components describing the four above mentioned subspaces of the shape variation were used for their formal comparison. The Euclidean distances of PC scores of each cell on the principal components occupying each subspace were evaluated by one-way repeated measures ANOVA followed by *post-hoc* Tukey’s pairwise range test implemented in PAST ver. 2.17c [50].

The analysis of the matching symmetry was based on the shape comparison of the terminal lobules of the lateral lobes, each described by the configuration of seven landmarks (Fig. 1c). The upper and lower lateral sublobes were analysed separately, because in our model species, *M. compereana*, they differ in their degree of lobulation. The lower lateral sublobe (LLS) has three additional branching levels, i.e. the terminal lobules are actually the 3^rd^-order lobules [20]. Conversely, the upper lateral sublobe (ULS) of *M. compereana* typically branches into four additional levels. Thus, the basic terminal units of the matching symmetry analysis in the ULS are the 4^th^-order lobules (Fig. 1). Before joint GPA and subsequent decomposition of symmetric and asymmetric components of the variation, correspondence among the lobules was achieved by sequential reflections of their landmark configurations across individual axes of symmetry. Thus, the configurations were reflected (and relabelled accordingly) across the axes differentiating the semicells, their “halves”, i.e. the quadrants, and the lobules within the quadrants. In the LLS analysis this last step included reflection and relabelling of the landmarks across the axis differentiating upper (e.g. *aa1* in Fig. 1c) and lower (*aa2*) terminal lobules. Likewise, two such symmetry axes were defined in the ULS analysis: symmetry across the main ULS incision (differentiating *ab1* from *ab4* and *ab2* from *ab3*) and symmetry across two minor incisions of ULS (*ab1* × *ab2* and *ab3* × *ab4*). Given two independent digitisations (for the assessment of measurement error), the resulting dataset comprised 1088 configurations for the LLS analysis and 2176 lobules in the ULS model.

The proportion of symmetric variation and individual asymmetric effects was quantified and evaluated in two separate multivariate non-parametric ANOVA models. The analyses were based on the matrices of tangent PDs among the landmark configurations of terminal lobules from LLS and ULS, respectively. In each analysis, the PD matrix was partitioned across the sources of variation (factors) by fitting a linear model. The factors were the individuals (cells) and all the asymmetric effects defined by multiple axes of symmetry intersecting the *Micrasterias* cell. The ANOVA model for the analysis of the morphometric symmetry and asymmetry has to be exhaustive, i.e. all the degrees of freedom (apart from the measurement error) have to be spanned by individual factors [44, 51]. In case of microalgal cells such as *Micrasterias*, the ambiguity of left-right and top-down correspondence among the specimens makes it impossible to include these factors as fixed effects, crossed with the main random effect of individuals, as is the case in Procrustes ANOVA models for symmetry analysis in multicellular organisms [44]. However, Klingenberg [1] suggested that in case of ambiguous correspondence of sides among specimens, the asymmetric factors should be included as effects nested within the individuals, and this is the approach used in the present study. As mentioned, factors describing individual subsets of the symmetric and asymmetric variation are mutually orthogonal. This means that they are uncorrelated in the ANOVA model. Even in the type I ANOVA with sequential calculation of sum of squares (SS), the order of the factors does not change the particular share of the variation they describe within the model.

**Fig. 2:**
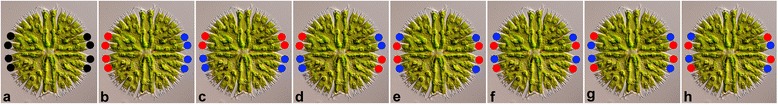
Components of matching symmetry and asymmetry among the 3^rd^-order terminal lobules in the lower lateral sublobe of cells. **a** Symmetry. **b** Left-right asymmetry. **c** Asymmetry across the isthmus axis. **d** Transversal asymmetry. **e** Intra-lobe asymmetry. **f** Intra-lobe and across-the-isthmus-axis asymmetry. **g** Intra-lobe and left-right asymmetry. **h** Intra-lobe and transversal asymmetry

In addition to the effects of individuals, the LLS ANOVA model comprised seven additional asymmetry effects (Fig. 2). The main effect of individuals in fact represented totally symmetric variation in shape of the terminal lobules (Fig. 2a). Under this effect, the shapes of all the lobules from each cell were averaged and the sum of squares related solely to differences in symmetric variation among the cells. The asymmetric effects, nested within the individuals, together spanned all the remaining degrees of freedom apart from the measurement error. First, there was an asymmetric factor highlighting the shape differences among the quadrants of each semicell (Fig. 2b). In this factor, the shapes of the quadrants were averaged in each cellular half according to the left-right axis and compared to the second half of each cell. The second asymmetric factor contrasted the lobules belonging to each semicell across the isthmus axis (Fig. 2c). Then, there was an asymmetric factor highlighting the transversal asymmetry according to both the above-mentioned axes of symmetry within the cells (Fig. 2d). The fourth asymmetric factor contrasted asymmetry in the shape of the lobules, positioned immediately by the isthmus axis, and the lobules adjacent to the incision between LLS and ULS (Fig. 2e). The final three asymmetric factors combined the asymmetry within the quadrants and across the horizontal (isthmus) or vertical (left-right) axes (Fig. 2f–h).

**Fig. 3:**
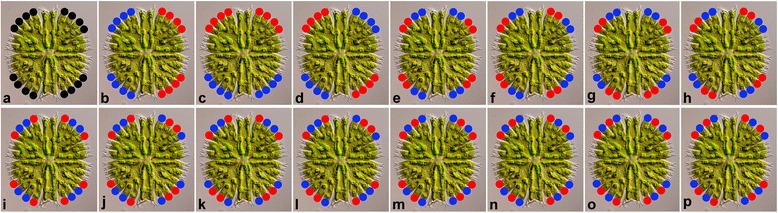
Components of matching symmetry and asymmetry among the 4^th^-order terminal lobules in the upper lateral sublobe of cells. **a** Symmetry. **b** Left-right asymmetry. **c** Asymmetry across the isthmus axis. **d** Transversal asymmetry. **e** Asymmetry between the 3^rd^-order lobules. **f** Asymmetry within the 3^rd^-order lobules (type I). **g** Inter-3^rd^-order-lobules and left-right asymmetry. **h** Inter-3^rd^-order-lobules and across-isthmus-axis asymmetry. **i** Inter-3^rd^-order-lobules and transversal asymmetry. **j** Intra-3^rd^-order-lobules (type I) and left-right asymmetry. **k** Intra-3^rd^-order-lobules (type I) and across-isthmus-axis asymmetry. **l** Intra-3^rd^-order-lobules (type I) and transversal asymmetry. **m** Asymmetry within the 3^rd^ order lobules (type II). **n** Intra-3^rd^-order-lobules (type II) and left-right asymmetry. **o** Intra-3^rd^-order-lobules (type II) and across-isthmus-axis asymmetry. **p** Intra-3^rd^-order-lobules (type II) and transversal asymmetry

The model for ULS terminal lobules included a total of 16 factors (Fig. 3). As in the LLS analysis, the effect of individuals represented symmetric variation of all 16 lobules within each cell and, thus, it illustrated mean differences in the shape of these lobules among the cells (Fig. 3a). Then, there were exactly 15 additional nested factors that combined the lobules into two equal groups within each cell and quantified the shape asymmetry spanned by these patterns. Once again, the factors included asymmetries across the left-right and isthmus axes (Fig. 3b,c), as well as their combination, yielding the transversally asymmetric pattern (Fig. 3d). Then, there were the within-quadrant asymmetric factors differentiating the lobules across the main incision of the ULS (Fig. 3e), as well as across the minor ULS incisions (Fig. 3i,m). The remaining nine asymmetric factors consisted of combinations among the asymmetries across one of the inter-quadrant axes (left-right, isthmus, transversal) and the intra-quadrant axes (Fig. 3f–h,j–l,n–p).

Multiple orthogonal asymmetric effects, nested within the individuals, required careful construction of the pseudo-F ratios that would reasonably compare the variation explained by a particular effect against the orthogonal components of the model. For the main effect of the individuals, the pseudo-F statistic was constructed as the ratio of the MS_ind_ and the error term formed by composite of the total intra-cell asymmetry, pooling sums of squares and degrees of freedom of all the components of the asymmetric variation [24]. Likewise, the particular asymmetric factors nested within the individuals were tested against the combination of the remaining asymmetric effects nested within the cells by pooling the SS and df of the asymmetric effects orthogonal to the evaluated factor (Additional file 2). Thus, the pseudo-F ratio of a particular asymmetric effect represented the relative contribution of that asymmetry pattern compared to the mean contribution of the remaining asymmetric effects, all of that nested within individual cells. The null hypothesis for the tests evaluating individual asymmetric factors was that a particular effect did not span more variation in the shape of the terminal lobules than a set of the remaining intracellular asymmetric effects.

The computations of the multivariate non-parametric ANOVA models were implemented by the functions *procD.lm* and *nested.update* of the package *geomorph*, ver. 3.0.0 [52], in R ver. 3.2.3. The function *procD.lm* performs statistical assessment of the factors included in the model by decomposition of a matrix of Procrustes distances among the objects (i.e., landmark configurations of individual terminal lobules) in a way which is equivalent to distance-based NPMANOVA. Significance of the factors was assessed by permutation tests with 999 repetitions that resulted in a random distribution of the F-values, which were adjusted to MS_effect_/MS_error_ in every random permutation. The *p*-values for individual effects were then estimated from resulting distribution of the random F-values as the percentiles of the effect sizes, i.e. the Z-scores, defined as standard deviations of the sampling distributions of the F-values [53, 54]. The degrees of freedom accounted for by individual effects were approximately equal (68 in case of the asymmetric components, 67 for symmetric variation). Therefore, the R^2^ values, computed as SS_effect_/SS_total_, could be used to compare the percentages of the total variation spanned by individual effects.

As a follow-up on the ANOVA models, I also conducted a *post-hoc* test based on the comparison of the mean tangent PDs among the corresponding lobules of the opposite semicells and among the cells. The test was designed to evaluate the null hypothesis that the corresponding lobules belonging to opposite semicells are not more similar in shape than the lobules belonging to different cells. For this test, the configurations of both corresponding lobules on a single semicell (in case of the most basal lobule, *aa1* and *ba1*) were averaged and compared to the corresponding configuration of the opposite semicell (an average of *ca1* and *da1*). Then, the tangent PDs between these configurations were computed for all 68 cells. Their mean represented the test value for this particular lobule. Likewise, the set of PDs among corresponding lobules belonging to different cells was acquired. A bootstrap distribution of mean among cell PDs was created by computing the mean of a random selection of 68 values from the intercellular set. This procedure was repeated 999 times. Finally, the intra-cell mean PD for each of the six terminal lobule positions was compared to the corresponding bootstrap distribution.

### Morphological integration

The two-block partial least squares analysis (PLS) was used to evaluate the degree of integration between the pairs of the terminal lobules. In geometric morphometrics, this analysis is also known as singular warps analysis [55]. It describes covariance between two morphometric datasets by extracting the singular axes that span their mutual patterns of covariation in shape [55, 56]. Thus, singular warps (SW) are the axes, resulting from the singular value decomposition of the matrix of covariances between two morphometric datasets. In an analogy to the principal component analysis, the first singular warp (SW1) describes the highest proportion of the covariation between both structures. Subsequent singular warps then successively span the remaining portions of the covariation. In other words, SW1 describes the variation in the first dataset that maximally explains the variation in the second one [56, 57]. Association between both axes of SW1 can be assessed by linear correlation analysis, yielding so-called PLS correlation values [51, 55]. The observed PLS correlation may be compared to a distribution of correlation values acquired by random permutation of the objects in one dataset in relation to the other. Significance of the PLS correlation means that the correlation coefficient between original singular axes was higher than the 95% percentile of the distribution obtained by the permutation procedure. In parallel, the RV coefficient was used as a measure of overall covariation between two sets of landmark configurations [58]. It can also be perceived as a multivariate generalization of the squared correlation coefficient (R^2^).

The analysis comprised 26 cellular landmark configurations. Each of them was again composed of seven landmarks and they represented individual terminal lobules of the lateral lobes and the apical parts of both polar lobes (Fig. 1c). Because the identity of individual quadrants is inherently ambiguous, the PLS correlation and RV coefficient values among mutually corresponding pairs were averaged. For example, this means that the PLS correlation between the lowest terminal lobule of LLS and the uppermost terminal lobule of ULS of the same quadrant was averaged from four values obtained from four separate runs of the singular warps analysis of *aa1* × *ab4*, *ba1* × *bb4*, *ca1* × *cb4*, and *da1* × *db4* (Fig. 1c). Allometry or size-dependent shape variation may be a strong integrating factor and thus can confound the integration patterns [59]. Therefore, the data were corrected for the allometric variation by a multivariate regression of shape on the centroid size of the objects. The residuals of the regression line were added to the consensus configuration so that resulting configurations represented shape variation that was not explained by size. The analyses of morphological integration were implemented using the functions *integration.test* in the *geomorph* package, ver. 3.0.0., and *morphol.integr* in *geomorph*, ver. 2.1.7. The significance of the PLS correlation values was assessed by randomisation tests with 999 random permutations. The test involved permuting the specimens in one data matrix relative to those in the second one and subsequent re-calculation of the PLS analysis for each iteration. Correlation between the matrices of of integration values evaluated by PLS correlation and RV coefficient was assessed by Mantel test in PAST, ver. 2.15 [50].

## Results

### Object symmetry

**Fig. 4:**
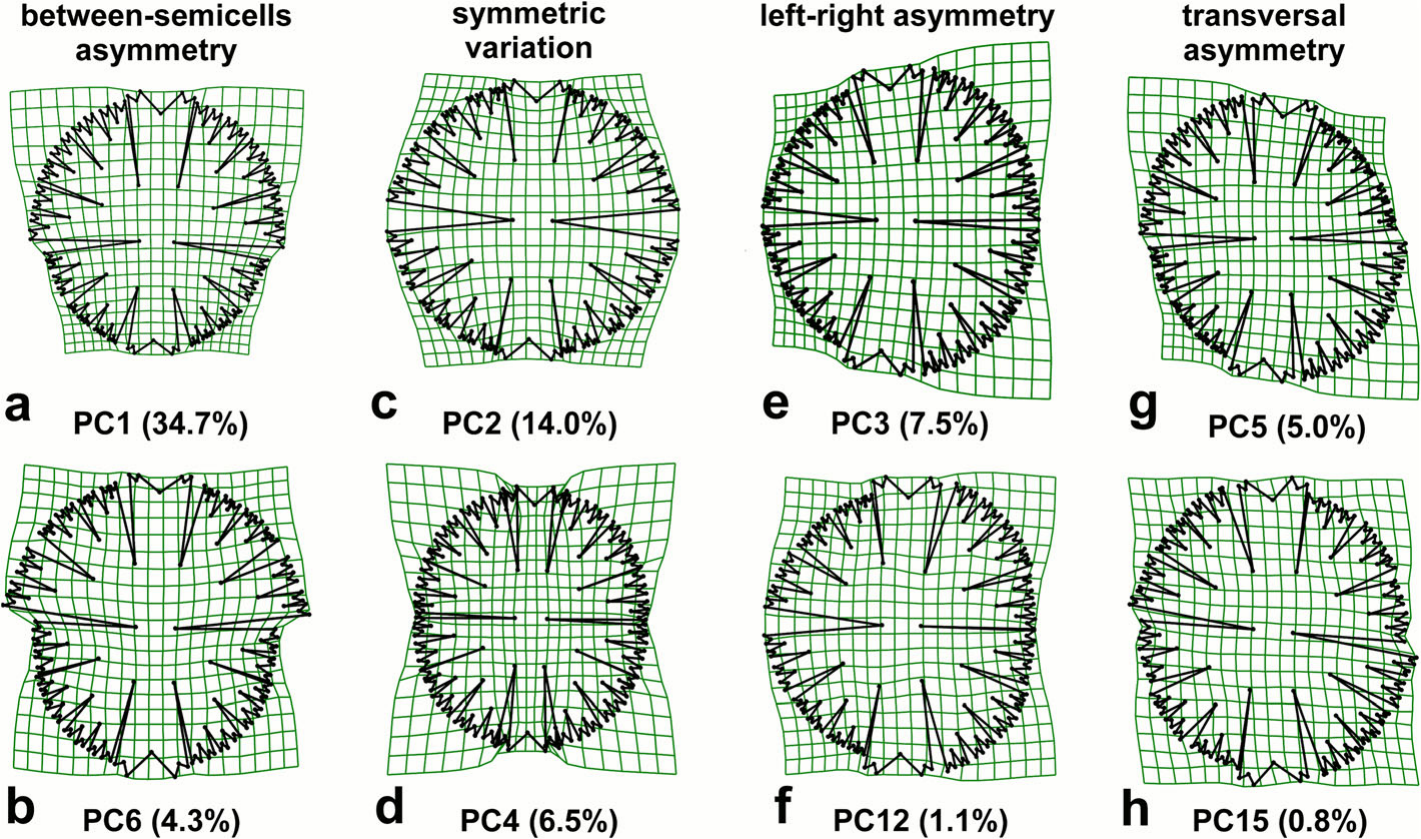
The thin-plate splines showing changes in cellular shapes spanned by PC axes of the principal component analysis decomposing patterns of biradial object symmetry. The two axes spanning most variation in each of the four symmetric and asymmetric subspaces are illustrated. **a**, **b** Asymmetry across the isthmus axis. **c**, **d** Totally symmetric variation. **e**, **f** Left-right asymmetry. **g**, **h** Transversal asymmetry combining shape differentiation across both axes

Asymmetry between semicells, i.e. across the isthmus axis, represented 48.9% of the total variation and proved to be the single most important pattern of the overall shape space. The first principal component (PC1) belonged to this subset of the shape space and it primarily described relative difference in size between both semicells (Fig. 4a). In addition, it emphasised the differences in width of the major incisions among the lobes, which were distinctly more opened in the smaller semicell. Interestingly, the pattern of variation related to PC1 also included differentiation in shape between both LLS terminal lobules. The most basal lobule of the smaller semicell was apparently more compressed than its adjacent counterpart. Conversely, both lobules of the larger semicell had a much more similar shape. PC6 was the second most important axis that belonged to the asymmetric subspace spanning the differentiation between the opposite semicells (Fig. 4b). It was also related to the difference in size of the semicells and width of the incisions. However, the relation between these two features was inverse to PC1, indicating that the larger semicell had more opened incisions separating the major lobes.

The purely symmetric subspace represented 31.4% of the total variation. Interestingly, the two most important axes in this subspace, PC2 and PC4, described variation patterns related to clearly different cellular parts. Variation across PC2 related mostly to shape changes in the lateral lobes (Fig. 4c), whereas variation across PC4 was characterised by shape variation of the polar lobes, as visualized by the grid compression in these areas (Fig. 4d). The left-right asymmetry represented 11.2% of the total variation. The most important axis belonging to this subspace, PC3, emphasised differences between the lateral lobes of each semicell (Fig. 4e). Likewise, PC12, the second most important axis with the left-right asymmetry pattern, described an inverse differentiation between the LLS and ULS of the lateral lobes (Fig. 4f). Finally, transversal asymmetry was the least represented component of the overall shape space with only 8.6% of the total variation. The most pronounced pattern belonging to this subspace was associated with PC5 and it spanned transversally arranged asymmetric differentiation among the lateral lobes (Fig. 4g). Conversely, PC15 largely described transversal asymmetry in the shape of the polar lobes (Fig. 4h).

Given the profound differences among the proportion of variation spanning each of the four symmetric and asymmetric subspaces, it is not surprising that the one-way ANOVA yielded a highly significant result, rejecting the hypothesis of their balanced occupation of the morphospace in the studied species (between groups SS = 0.0093, df = 3, MS = 0.0031, F = 56.2, *p* = 2.05 × 10^−26^). The *post-hoc* Tukey’s pairwise comparisons revealed that two dominant subspaces (symmetry and inter-semicell asymmetry) were significantly overrepresented in the overall shape space compared to both remaining subspaces, i.e. the left-right and transversal asymmetry (Table 1). The purely symmetric component of the variation was significantly less represented that the inter-semicell asymmetry, albeit with the p-value of 0.012. Conversely, the differences between the two minor subspaces in their proportion of the total variation proved to be insignificant.

**Table 1:** Proportions of variance in shape of the cells accounted for by different components of biradial object symmetry

One-way repeated measures ANOVA				
	SS	df	MS	F / p-value
Between components	0.0093	3	0.0031	56.2 / 2.05 × 10^−26^
Within components	0.0162	268	6.05 × 10^−5^	
Between individuals	0.0051	67	7.68 × 10^−5^	
Total	0.0255	271		
Tukey’s pairwise comparisons (Q statistic/*p*-value)				
	symmetry	asymmetry between semicells	left-right asymmetry	transversal asymmetry
Symmetry		0.012	7.72 × 10^−6^	7.72 × 10^−6^
Asymmetry between semicells	4.31		7.72 × 10^−6^	7.72 × 10^−6^
Left-right asymmetry	6.90	14.20		0.867
Transversal asymmetry	10.99	15.29	1.09	

### Matching symmetry

The Procrustes ANOVA model of the shape variation among the LLS terminal lobules included a single factor differentiating the individuals and seven asymmetric effects nested within the cells (Table 2). Three of these eight effects described the majority of the observed variation. First, differences among the individuals, based on the average configurations of the lobules from each cell, exhibited 23.8% of the total variation, a proportion that proved to be highly significant when tested against the total asymmetry. Two asymmetric effects were significant as well. The asymmetry across the isthmus axis, differentiating between the lobules from opposite semicells (Fig. 2c), accounted for 21.2% of the variation, roughly comparable to the variation among the individuals. In addition, asymmetric variation between the lobules of each quadrant (Fig. 2e), averaged within the cells, accounted for 18.0% of the variation, a highly significant proportion when tested against the remaining asymmetric components. The combination of these two asymmetric effects (Fig. 2f) described 10.9% of the total variation within the model. However, the mean squares (MS) of this effect divided by MS of the pooled asymmetry of the remaining components yielded an F-ratio of 1.08 that did not prove significant in the randomisation test. Other components, including the left-right and transversal asymmetry, yielded F-ratios less than 1, which means that there was, on average, more variance explained by other asymmetric effects than by any of these components.

**Table 2:** Procrustes ANOVA evaluating symmetric and asymmetric variation among the LLS terminal lobules of *Micrasterias compereana*

Source	Df	SS	MS	R^2^	F	Z	*p*-value	Fig
Individual	67	5.20	0.078	0.238	2.36	1.78	0.001	2a
Left-right asymmetry (ind)	68	1.19	0.018	0.055	0.49	0.46	1.000	2b
Inter-semicell asymmetry (ind)	68	4.62	0.068	0.212	2.52	1.81	0.001	2c
Transversal asymmetry (ind)	68	1.36	0.019	0.062	0.57	0.52	1.000	2d
Intra-lobe asymmetry (ind)	68	3.94	0.058	0.180	2.02	1.53	0.001	2e
Intra-lobe and Left-right (ind)	68	1.04	0.015	0.048	0.43	0.40	1.000	2f
Intra-lobe and Inter-semicell (ind)	68	2.38	0.035	0.109	1.08	0.92	0.799	2g
Intra-lobe and Transversal (ind)	68	1.10	0.016	0.050	0.45	0.42	1.000	2h
[Total asymmetry nested within individuals]	476	15.64	0.032	0.716				
Measurement error	544	0.99	0.002	0.046				
Total	1087	21.83						

*Df* degrees of freedom, *SS* sum of squares, *MS* mean squares, *R* ^*2*^ percentage of variance explained by the effect, *F* pseudo-F ratio, *Z* effect size, *p-value* percentile of the effect size in the random distribution of F values, *Fig* figure illustrating particular (a) symmetric patterns

The ULS Procrustes ANOVA model comprised symmetric variation among the individuals and 15 nested asymmetric effects. As in the LLS model, three of these 16 effects proved to be dominant with regard to their share of the total variation (Table 3). With 23.2% of the variation in shape, the differences among the individuals were highly significant against the total asymmetry nested within the cells. Among the nested effects, asymmetry between the opposite semicells was also highly significant with 16.4% of the total variation within the model (Fig. 3c). Finally, a particular type of asymmetry across the minor ULS incisions (Fig. 3i) accounted for 13.2% of the variation and was considerably more important than all the other asymmetric components combined. In addition, it proved to be significant when tested against the pooled sum of squares and degrees of freedom of remaining asymmetric components. Other components of asymmetry were considerably less represented and they yielded insignificant F-ratios lower or only slightly higher than 1 (in case of left-right and transversal asymmetric effects).

**Table 3:** Procrustes ANOVA evaluating symmetric and asymmetric variation among the ULS terminal lobules of *Micrasterias compereana*

Source	Df	SS	MS	R^2^	F	Z	*p*-value	Fig
Individual	67	12.69	0.189	0.232	5.01	3.80	0.001	3a
Left-right asymmetry (ind)	68	2.86	0.042	0.052	1.12	1.04	0.253	3b
Inter-semicell asymmetry (ind)	68	8.98	0.132	0.164	4.25	3.30	0.001	3c
Transversal asymmetry (ind)	68	2.61	0.038	0.048	1.02	0.95	0.703	3d
Inter-3^rd^-order-lobules asymmetry (ind)	68	1.89	0.028	0.035	0.72	0.68	1.000	3e
Inter-3^rd^-order-lobules and Left-right (ind)	68	1.19	0.018	0.022	0.45	0.43	1.000	3f
Inter-3^rd^-order-lobules and Inter-semicell (ind)	68	1.42	0.021	0.026	0.54	0.52	1.000	3g
Between-3^rd^-order-lobules and Transversal (ind)	68	0.99	0.015	0.018	0.37	0.36	1.000	3h
Intra-3^rd^-order-lobules asymmetry - type I (ind)	68	7.19	0.106	0.132	3.21	2.65	0.001	3i
Intra-3^rd^-order-lobules–type I and Left-right (ind)	68	1.67	0.025	0.031	0.63	0.61	1.000	3j
Intra-3^rd^-order-lobules–type I and Inter-semicell (ind)	68	2.37	0.035	0.043	0.92	0.87	0.963	3k
Intra-3^rd^-order-lobules–type I and Transversal (ind)	68	1.73	0.025	0.032	0.66	0.63	1.000	3l
Intra-3^rd^-order-lobules-asymmetry–type II (ind)	68	1.98	0.029	0.036	0.76	0.72	1.000	3m
Intra-3^rd^-order-lobules–type II and Left-right (ind)	68	1.09	0.016	0.019	0.41	0.39	1.000	3n
Intra-3^rd^-order-lobules–type II and Inter-semicell (ind)	68	1.52	0.022	0.028	0.58	0.55	1.000	3o
Intra-3^rd^-order-lobules–type II and Transversal (ind)	68	1.06	0.016	0.019	0.39	0.38	1.000	3p
[Total asymmetry nested within individuals]	1020	38.56	0.038	0.705				
Measurement error	1088	3.40	0.003	0.062				
Total	2175	54.66						

*Df* degrees of freedom, *SS* sum of squares, *MS* mean squares, *R* ^*2*^ percentage of variance explained by the effect, *F* pseudo-F ratio, *Z* effect size, *p-value* percentile of the effect size in the random distribution of F values, *Fig* figure illustrating particular (a)symmetric patterns

### The *post-hoc* test on Procrustes distances

**Fig. 5:**
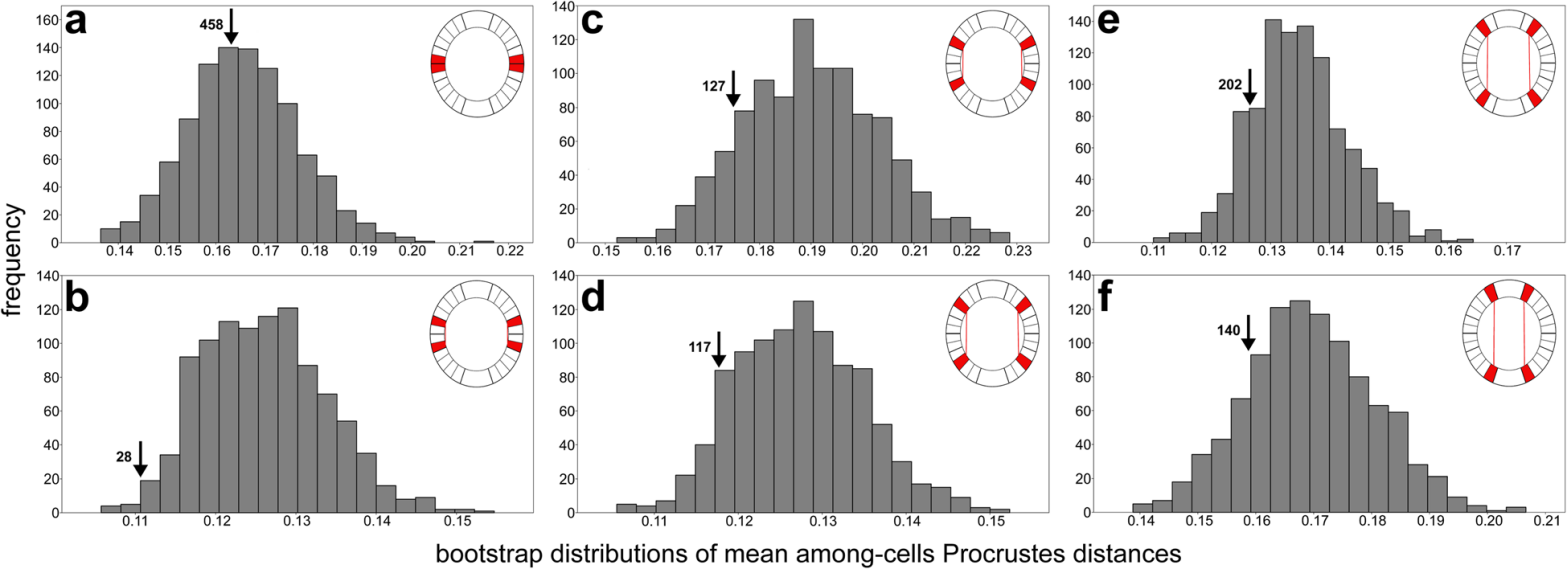
Bootstrap distributions based on 999 mean PDs among the corresponding lobules from different cells. Arrows indicate the positions and ranks of the mean intra-cell PDs evaluating the shape differences among the corresponding lobules from the opposite semicells. **a**, **b** The 3^rd^ order lobules of LLS. **c–f** The 4^th^ order lobules of ULS

In both LLS and ULS, a substantial part of the variation was apportioned to asymmetry between the lobules belonging to the opposite semicells. In fact, this effect proved to be the single most important asymmetric component in both models. In addition, in the analysis of the object symmetry, the asymmetric variation also proved to be dominant across the four symmetric and asymmetric subspaces, highlighting the importance of differences across the isthmus axis. Thus, the *post-hoc* tests evaluated the hypothesis that the semicells may actually be completely independent with regard to the shape of the terminal lobules. In all six lobule positions, the mean PD within the cells was lower than the mean value of the inter-cell bootstrap distribution (Fig. 5). The opposite most basal lobules (i.e. *aa1* vs. *da1*) were most dissimilar, as the intra-cell mean PD was about the same as the average values of the inter-cell bootstrap distribution with 458 random sets having lower mean PD than the observed value (Fig. 5a). Conversely, the adjacent lobule (*aa2* vs. *da2*) had a significantly lower mean intra-cell PD value than that with the bootstrap distribution. Only 28 random sets out of the 999 taken from the inter-cell shape comparisons yielded a lower mean PD (Fig. 5b). All the mean intra-cell PDs of the four ULS lobules fell in the lowest quarter of the bootstrap distribution with 117 to 202 random sets yielding lower inter-cell mean PDs than the respective observed values of the intra-cell sets (Fig. 5c–f).

### Morphological integration

**Fig. 6:**
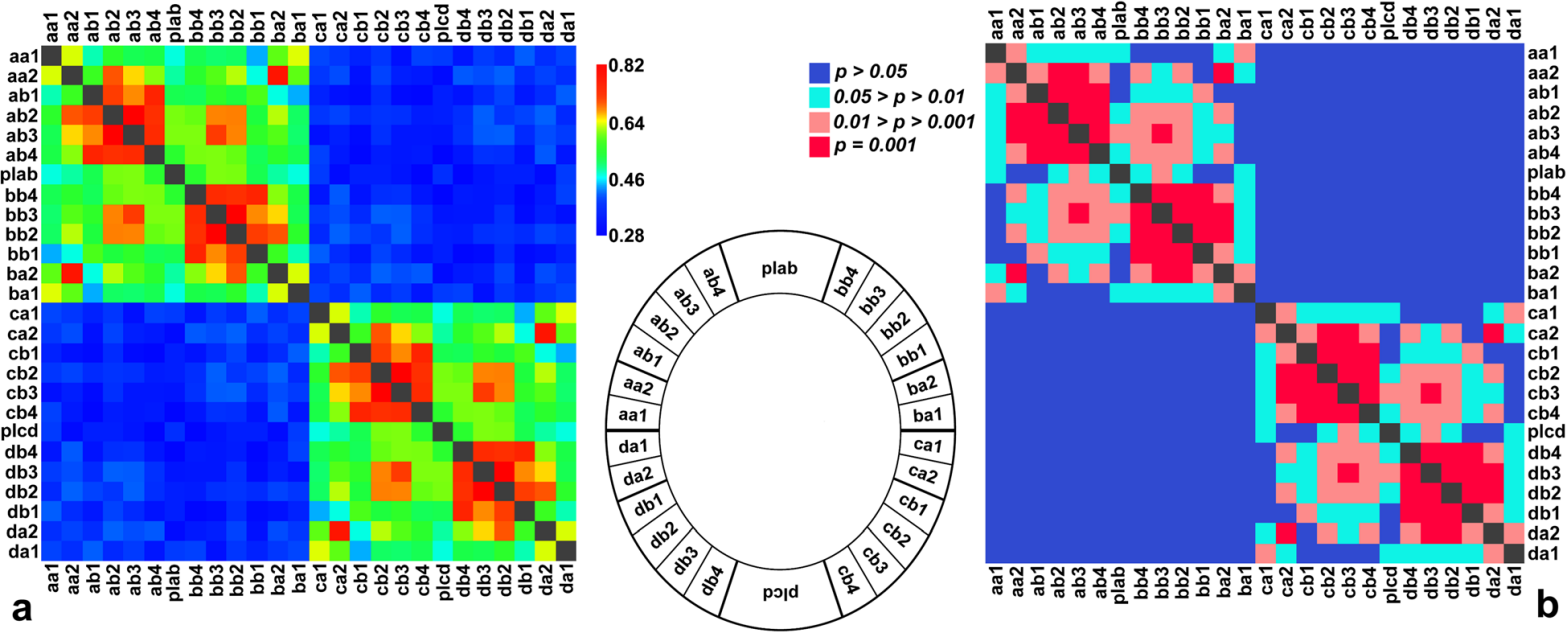
Results of the PLS analyses evaluating morphological integration among the terminal lobules of cells. The position of individual lobules on cells is illustrated by a simplified scheme of the cellular morphology. **a** Correlation values between the first pair of the singular warps in each PLS analysis. **b** The p-values resulting from the permutation tests of the PLS correlations

The PLS correlation among the lobules varied substantially from 0.28 to 0.82 in different pairs (Fig. 6a, Additional file 3). Likewise, the RV coefficient varied from 0.05 to 0.58 (Additional file 3). The matrix correlation between the test values from both analyses were very high (Mantel *r* = 0.97, *p* < 0.001), indicating that they illustrated closely similar patterns of the morphological integration among the lobules. The most striking pattern was that of the differences in integration levels among the lobules belonging to a single semicell in comparison to those of the opposite semicells. In fact, not a single pair of the lobules from the opposite semicells reached significant covariance levels (Fig. 6b). The opposite polar lobes were also mutually not integrated. Conversely, all the pairs of lobules within the semicells had higher integration values than the lobules from the opposite semicells. The most tightly integrated lobules were those forming a joint ULS. Conversely, morphological integration between two lobules forming the LLS was markedly lower. Integration among the lobules from opposite quadrants was generally lower, but the mutually corresponding lobules (*ab1* × *bb1*) were more strongly integrated, reaching the integration levels typical for intra-quadrant comparisons. The polar lobe was weakly integrated with the lobules of the adjacent lateral lobes. Interestingly, the lobules that were spatially more distant of LLS were also more independent of the polar lobe variation, whereas the ULS lobules had modest but still slightly higher levels of covariance with the polar lobe.

## Discussion

The analyses demonstrated that there are two dominant types of morphological asymmetry among the terminal lobules of the cells. Asymmetry between the semicells across the isthmus axis was consistently detected as the single most important asymmetric effect in all the analyses, such as decomposition of the object symmetry and two parallel analyses of the matching symmetry. This was further supported by the tests evaluating mean PDs among the lobules within and among the cells. Comparison of the mean intra-cell PDs with the bootstrap distribution of mean inter-cell PDs showed that shape differences among the opposite lobules could be exceeded with relatively large odds by a randomly created dataset of PDs among the corresponding lobules from different cells. A single exception was the upper LLS lobule (the *aa2* position), where just 2.8% of the mean inter-cell PDs were larger than the original intra-cells value. Thus, in these particular lobules, the shape in opposite semicells was unlikely to be entirely independent. However, whether this particular cellular part may really be systematically different from other terminal lobules in its shape asymmetry across the isthmus axis cannot be concluded based on the marginally significant result of the present analysis. This particular question would require additional investigation in other populations and species of the *Micrasterias* lineage.

Relative morphogenetic independence of the terminal lobules in the opposite semicells was further supported by the absence of significant covariance in shape in the studied population. These tests showed that *Micrasterias* cells are composed of two independent parts, without any significant covariance among their lobules. These results concur with previous microscopic observations of Waris, Kallio, and Lehtonen [28–30], who demonstrated that morphogenesis of the major parts of a developing semicell, such as entire lateral lobes in uniradiate or aradiate mutants, could be affected by the morphology of their older opposite counterparts. However, they did not observe any influence at the level of terminal lobules in normally developing cells. In addition, the morphological integration patterns within the cells indicated that the polar lobes constitute more or less independent morphogenetic units, i.e. cellular modules. This also concurs with earlier observations, which illustrated that in developing semicells, the morphogenesis of the polar lobe slightly lags behind both lateral lobes [16]. Moreover, the development of the lateral lobes could be disrupted with no effects on the morphogenesis of the polar lobes [30].

Conversely, the lateral lobes within a single semicell did not prove to be morphogenetically independent. Especially the lobules situated in the same position within their respective lateral lobes were relatively tightly integrated. A possible link between both lateral lobes has previously been illustrated by Gärtner and Meindl [31], who showed that uniradiate mutants, i.e. cells lacking one lateral lobe in each semicell, tend to gradually return to their original, wild type morphology with two lateral lobes adjacent to the centrally located polar lobe. There is apparently at least one lineage of the *Micrasterias* clade that consists of species lacking both lateral lobes, formerly known as the traditionally defined genus *Triploceras* [23, 60]. It has been speculated that this lineage might have arisen by permanent blockage of the morphogenesis in both lateral lobes [23]. Conversely, there seem to be not a single species of the *Micrasterias* clade possessing only one lateral lobe. Thus, mutual interconnection of the developmental processes between both lateral lobes, as illustrated here by the tests of morphological integration and other experimental observations [31], may prevent evolutionary radiation based on the uniradiate morphs. The integration levels among the lobules from the two sublobes of each lateral lobe (LLS and ULS) were also considerably different. This was most apparent in four lobules from ULS, which were mutually tightly integrated, but their relation to the LLS lobules, as well as to the lobules from the adjacent quadrant, was weak. This pattern generally confirmed the characteristics of the theoretical models of the *Micrasterias* cytomorphogenesis [18, 41, 42]. These models presumed that the growth patterns of the developing semicell are compartmentalised and separate parts of the structure may develop relatively independently. In late stages of the morphogenetic process, when the terminal lobules are formed, tip growth and patterning occurs in multiple active centres which may only be minimally coordinated [42].

Such pattern could obviously also include changes in mutual integration levels among developing lobes during semicell ontogenesis. Hallgrímsson and his colleagues introduced a metaphor of a “developmental palimpsest” for description of mutual changes in the integration patterns among organismal parts during morphogenesis [61]. In *Micrasterias* semicells this could include gradual separation of individual developing lobes and lobules which could become less integrated with increasing number of lobe branching on cells. Relatively weak but significant integration among more distant parts in mature semicells could then be explained as residue of their tigher relation in earlier stages of the development. Such hypothesis could be tested by evaluating the developmental integration, i.e. by tracking developement stages of individual semicells placed in experimental microchambers.

The second most important asymmetric effect detected in this study involved shape differentiation among the lobules of the same lateral lobe. The MS values for this pattern of asymmetry were approximately 3 to 6 times higher than those for other asymmetric components, save for the inter-semicell asymmetry. In general, this effect implied that adjacent terminal lobules differed in shape in a manner shared by all four cellular quadrants. While the inter-semicell aymmetry may apparently be related to the diachronic morphogenesis of the semicells, which may take place in locally different environmental conditions [12, 27], asymmetry among the adjacent lobules within the quadrants may be increased by species-specific morphogenetic factors; that is, by phylogenetically fixed patterns of asymmetric differentiation of sublobes and lobules of cells. The model species in this study, *M. compereana*, belongs to clade C of the *Micrasterias* lineage, which in contrast to most other members of the genus has distinctly asymmetric shapes of the LLS and ULS [21, 23, 62]. Therefore, asymmetry among the terminal lobules can perhaps be viewed as a continuation of this pattern to all levels of cellular branching. Interestingly, the mathematical model of cellular morphogenesis [17, 18], which, under varying conditions, leads to 3-D shapes closely resembling various *Micrasterias* and *Euastrum* species, was also able to simulate asymmetric branching of ULS and LLS, similar to the patterns observed in species of the clade C, such as *M. rotata* or *M. compereana* [18]. This was due to the concentration gradient of the theoretical patterning compound from the pole of the developing semicell to its bases, which led to earlier branching of the ULS [18]. If asymmetry between the adjacent lobes is shared down the branching order, as indicated by the present analysis of the morphological asymmetry, it may imply that additional mechanisms are breaking dichotomy of the branching process. It has been shown that the pattern-forming processes in desmid cells occur at the plasma membrane of the developing semicells [13, 33, 63]. Thus, systematic morphological asymmetry between the adjacent terminal lobules should be preceded by uneven distribution of the Ca^2+^ channels and other membrane proteins, such as receptors for vesicle membranes, which are considered responsible for pattern formation during semicell growth, resulting in differences in their final morphology [14, 40].

It should be noted that asymmetric lobules within the lateral lobes can be unambiguously assigned across the axis of symmetry with respect to their position in the lateral lobe. Therefore, this asymmetry could potentially be decomposed into the genetically fixed directional asymmetry, and individual asymmetric deviations from this pattern. The deviations from mean asymmetry could then in future studies be tested against a suite of abiotic factors, such as temperature, nutrients, or toxins. Its increased levels in particular populations of a studied species might indicate a role for developmental stress in the morphogenesis of the semicells. In organisms inhabiting water ecosystems, increased developmental instability resulting in asymmetric deviations in morphology of the symmetric body parts has been found to strongly correlate with higher concentrations of environmental stressors, such as organic and inorganic pollutants [64–66]. In *Micrasterias* species, such analysis could be particularly intriguing with regard to known toxic effects of certain heavy metals such as Cd, Cr, or Al on intracellular architecture, metabolism, and cytomorphogenesis [67–69]. In addition, these toxic ions are typically more soluble in the low pH conditions of the acidic wetlands, which constitute a typical desmid habitat [21, 60].

## Conclusions

In summary, this study illustrates that *Micrasterias* cells are composed of parts with widely different levels of morphological integration. These differences in integration among the cellular regions play a key role in the evolution of their shape, especially with regard to the relative independence of the polar lobe and a pair of the lateral lobes within a single semicell. The integration patterns were also mirrored by the results of the shape asymmetry analyses. It has been shown that asymmetry among the terminal lobules of cells is largely explained by two major patterns. The first is related to a high degree of independence and shape differences between the two cellular halves (semicells). It has been showed that opposite terminal lobules belonging to the opposite semicells are typically not more similar than the lobules from different cells. This indicates that morphogenetic processes leading to shapes of terminal lobules in opposite semicells may actually be completely independent. The second asymmetric pattern describes the differentiation between the adjacent lobules. This prominent pattern of morphological asymmetry may have implications for modelling cellular morphogenesis. In addition, it can be further investigated in future studies focusing on both the evolutionary structure of the entire *Micrasterias* lineage, as well as on environmental stress factors that may destabilise cellular development. Finally, the study showed that application of the methodological kits of geometric morphometrics may complement cell biology studies concentrated on the intracellular and genetic mechanisms of morphogenesis, as well as the theoretical studies modelling the patterns of the cell shape development. A combination of these profoundly different but complementary scientific approaches may reassert the position of the desmids as a prime model group for the research into the evolutionary patterns of cellular morphology.

## Additional files

Additional file 1: Coordinates of 208 landmarks depicted on cells of *Micrasterias compereana.* (TXT 231 kb)
Additional file 2: Formulas for the degrees of freedom, mean squares, and pseudo-F values in the Procrustes ANOVA models decomposing matching symmetry in LLS and ULS terminal lobules. (PDF 81 kb)
Additional file 3: PLS correlations among the terminal lobules of *M. compereana* cells and their significance values. (PDF 302 kb)
