# Supplementary material for: Asymmetry and integration of cellular morphology in Micrasterias compereana
Source: BMC Evol Biol. 2017 Jan 3;17:1. doi: 10.1186/s12862-016-0855-1 (PMC5209845; doi:10.1186/s12862-016-0855-1)
Supplement: Additional file 2: — Formulas for the degrees of freedom, mean squares, and pseudo-F values in the Procrustes ANOVA models decomposing matching symmetry in LLS and ULS terminal lobules. (PDF 81 kb) [file 12862_2016_855_MOESM2_ESM.pdf]

Additional file 2 Formulas for the degrees of freedom, mean squares, and pseudo-F values in the Procrustes ANOVA models decomposing matching symmetry in LLS and ULS terminal lobules

LLS (lower lateral sublobe)

Source	Abbreviation	Df	MS	Pseudo-F ratio
Individual	ind	$I-1$	$SS_{ind}/I-1$	$MS_{ind}/MS_{lrt:bet:wlb(ind)}$
Left-right asymmetry (ind)	wit	$I(J-1)$	$SS_{lrt(ind)}/I(J-1)$	$MS_{lrt(ind)}/MS_{bet:wlb(ind)}$
Inter-semicell asymmetry (ind)	bet	$I(J-1)$	$SS_{bet(ind)}/I(J-1)$	$MS_{bet(ind)}/MS_{lrt:wlb(ind)}$
Transversal asymmetry (ind)	trn	$I(J-1)$	$SS_{trn(ind)}/I(J-1)$	$MS_{trn(ind)}/MS_{bet:wlb(ind)}$
Intra-lobe asymmetry (ind)	wlb	$I(J-1)$	$SS_{wlb(ind)}/I(J-1)$	$MS_{wlb(ind)}/MS_{bet:lrt(ind)}$
Intra-lobe and Left-right (ind)	wwt	$I(J-1)$	$SS_{wlt(ind)}/I(J-1)$	$MS_{wlt(ind)}/MS_{bet:lrt(ind)}$
Intra-lobe and Inter-semicell (ind)	wbt	$I(J-1)$	$SS_{wbt(ind)}/I(J-1)$	$MS_{wbt(ind)}/MS_{bet:lrt(ind)}$
Intra- lobe and Transversal (ind)	wtr	$I(J-1)$	$SS_{wtr(ind)}/I(J-1)$	$MS_{wtr(ind)}/MS_{bet:lrt(ind)}$
Measurement error	mre	$IJ^3(r-1)$	$SS_{mre}/IJ^3(r-1)$	
Total		IJ^3r-1		

ULS (upper lateral sublobe)

Source	Abbrev.	Df	MS	Pseudo-F ratio
Individual	ind	$I-1$	$SS_{ind}/I-1$	$MS_{ind}/MS_{lrt:bet:blb:wlb(ind)}$
Left-right asymmetry (ind)	lrt	$I(J-1)$	$SS_{lrt(ind)}/I(J-1)$	$MS_{lrt(ind)}/MS_{bet:blb:wlb(ind)}$
Inter-semicell asymmetry (ind)	bet	$I(J-1)$	$SS_{bet(ind)}/I(J-1)$	$MS_{bet(ind)}/MS_{lrt:blb:wlb(ind)}$
Transversal asymmetry (ind)	trn	$I(J-1)$	$SS_{trn(ind)}/I(J-1)$	$MS_{trn(ind)}/MS_{bet:blb:wlb(ind)}$
Inter-3rd-order-lobules asymmetry (ind)	blb	$I(J-1)$	$SS_{blb(ind)}/I(J-1)$	$MS_{blb(ind)}/MS_{bet:lrt:wlb(ind)}$
Inter-3rd-order-lobules and Left-right (ind)	bll	$I(J-1)$	$SS_{bll(ind)}/I(J-1)$	$MS_{bll(ind)}/MS_{lrt:bet:wlb(ind)}$
Inter-3rd-order-lobules and Inter-semicell (ind)	bbl	$I(J-1)$	$SS_{bbl(ind)}/I(J-1)$	$MS_{bbl(ind)}/MS_{lrt:bet:wlb(ind)}$
Inter-3rd-order-lobules and Transversal (ind)	btr	$I(J-1)$	$SS_{btr(ind)}/I(J-1)$	$MS_{btr(ind)}/MS_{lrt:bet:wlb(ind)}$
Intra-3rd-order-lobules asymmetry – type I (ind)	wlb	$I(J-1)$	$SS_{wlb(ind)}/I(J-1)$	$MS_{wlb(ind)}/MS_{bet:lrt:blb(ind)}$
Intra-3rd-order-lobules – type I and Left-right (ind)	wlr	$I(J-1)$	$SS_{wlr(ind)}/I(J-1)$	$MS_{wlr(ind)}/MS_{lrt:bet:blb(ind)}$
Intra-3rd-order-lobules – type I and Inter-semicell (ind)	wbl	$I(J-1)$	$SS_{wbl(ind)}/I(J-1)$	$MS_{wbl(ind)}/MS_{lrt:bet:blb(ind)}$
Intra-3rd-order-lobules – type I and Transversal (ind)	wtr	$I(J-1)$	$SS_{wtr(ind)}/I(J-1)$	$MS_{wtr(ind)}/MS_{lrt:bet:blb(ind)}$
Intra-3rd-order-lobules asymmetry - type II (ind)	blw	$I(J-1)$	$SS_{blw(ind)}/I(J-1)$	$MS_{blw(ind)}/MS_{lrt:bet:wlb(ind)}$
Intra-3rd-order-lobules – type II and Left-right (ind)	bwl	$I(J-1)$	$SS_{bwl(ind)}/I(J-1)$	$MS_{bwl(ind)}/MS_{lrt:bet:wlb(ind)}$
Intra-3rd-order-lobules – type II and Inter-semicell (ind)	bwb	$I(J-1)$	$SS_{bwb(ind)}/I(J-1)$	$MS_{bwb(ind)}/MS_{lrt:bet:wlb(ind)}$
Intra-3rd-order-lobules – type II and Transversal (ind)	bwt	$I(J-1)$	$SS_{bwt(ind)}/I(J-1)$	$MS_{bwt(ind)}/MS_{lrt:bet:wlb(ind)}$
Measurement error		$IJ^4(-1)$	$SS_{mre}/IJ^4(r-1)$	
Total		IJ^4r-1		

I – number of individuals (I=68), J – number of levels in the asymmetric effects (J=2), r – number of repetitions (r=2)
