# Supplementary material for: Asymmetry and integration of cellular morphology in Micrasterias compereana
Source: BMC Evol Biol. 2017 Jan 3;17:1. doi: 10.1186/s12862-016-0855-1 (PMC5209845; doi:10.1186/s12862-016-0855-1)
Supplement: Additional file 3: — PLS correlations among the terminal lobules of M. compereana cells and their significance values. (PDF 302 kb) [file 12862_2016_855_MOESM3_ESM.pdf]

Additional file 3 PLS correlations among the terminal lobules of *M. compereana* cells, their significance values, the squared singular values for first and second pairs of singular warps expressed as percentages of the sum of squared covariances and RV coefficients among the terminal lobules

PLS correlations

	aa1	aa2	ab1	ab2	ab3	ab4	plab	bb4	bb3	bb2	bb1	ba2	ba1	ca1	ca2	cb1	cb2	cb3	cb4	plcd	db4	db3	db2	db1	da2	da1
**aa1**	x	0.630	0.480	0.549	0.513	0.519	0.469	0.513	0.502	0.506	0.439	0.592	0.631	0.399	0.385	0.352	0.384	0.319	0.395	0.390	0.331	0.390	0.364	0.412	0.372	0.397
**aa2**	0.630	x	0.568	0.693	0.650	0.608	0.485	0.545	0.587	0.621	0.467	0.771	0.592	0.385	0.403	0.321	0.397	0.348	0.337	0.308	0.409	0.404	0.413	0.369	0.395	0.372
**ab1**	0.480	0.568	x	0.708	0.672	0.745	0.514	0.510	0.554	0.526	0.582	0.467	0.439	0.352	0.321	0.307	0.366	0.309	0.298	0.347	0.326	0.365	0.397	0.335	0.369	0.412
**ab2**	0.549	0.693	0.708	x	0.820	0.724	0.602	0.596	0.674	0.675	0.526	0.621	0.506	0.384	0.397	0.366	0.382	0.375	0.343	0.347	0.372	0.412	0.384	0.397	0.413	0.364
**ab3**	0.513	0.650	0.672	0.820	x	0.734	0.603	0.604	0.710	0.674	0.554	0.587	0.502	0.319	0.348	0.309	0.375	0.334	0.333	0.331	0.378	0.410	0.412	0.365	0.404	0.390
**ab4**	0.519	0.608	0.745	0.724	0.734	x	0.545	0.602	0.604	0.596	0.510	0.545	0.513	0.395	0.337	0.298	0.343	0.333	0.339	0.294	0.332	0.378	0.372	0.326	0.409	0.331
**plab**	0.469	0.485	0.514	0.602	0.603	0.545	x	0.545	0.603	0.602	0.514	0.485	0.469	0.390	0.308	0.347	0.347	0.331	0.294	0.374	0.294	0.331	0.347	0.347	0.308	0.390
**bb4**	0.513	0.545	0.510	0.596	0.604	0.602	0.545	x	0.734	0.724	0.745	0.608	0.519	0.331	0.409	0.326	0.372	0.378	0.332	0.294	0.339	0.333	0.343	0.298	0.337	0.395
**bb3**	0.502	0.587	0.554	0.674	0.710	0.604	0.603	0.734	x	0.820	0.672	0.650	0.513	0.390	0.404	0.365	0.412	0.410	0.378	0.331	0.333	0.334	0.375	0.309	0.348	0.319
**bb2**	0.506	0.621	0.526	0.675	0.674	0.596	0.602	0.724	0.820	x	0.708	0.693	0.549	0.364	0.413	0.397	0.384	0.412	0.372	0.347	0.343	0.375	0.382	0.366	0.397	0.384
**bb1**	0.439	0.467	0.582	0.526	0.554	0.510	0.514	0.745	0.672	0.708	x	0.568	0.480	0.412	0.369	0.335	0.397	0.365	0.326	0.347	0.298	0.309	0.366	0.307	0.321	0.352
**ba2**	0.592	0.771	0.467	0.621	0.587	0.545	0.485	0.608	0.650	0.693	0.568	x	0.630	0.372	0.395	0.369	0.413	0.404	0.409	0.308	0.337	0.348	0.397	0.321	0.403	0.385
**ba1**	0.631	0.592	0.439	0.506	0.502	0.513	0.469	0.519	0.513	0.549	0.480	0.630	x	0.397	0.372	0.412	0.364	0.390	0.331	0.390	0.395	0.319	0.384	0.352	0.385	0.399
**ca1**	0.399	0.385	0.352	0.384	0.319	0.395	0.390	0.331	0.390	0.364	0.412	0.372	0.397	x	0.630	0.480	0.549	0.513	0.519	0.469	0.513	0.502	0.506	0.439	0.592	0.631
**ca2**	0.385	0.403	0.321	0.397	0.348	0.337	0.308	0.409	0.404	0.413	0.369	0.395	0.372	0.630	x	0.568	0.693	0.650	0.608	0.485	0.545	0.587	0.621	0.467	0.771	0.592
**cb1**	0.352	0.321	0.307	0.366	0.309	0.298	0.347	0.326	0.365	0.397	0.335	0.369	0.412	0.480	0.568	x	0.708	0.672	0.745	0.514	0.510	0.554	0.526	0.582	0.467	0.439
**cb2**	0.384	0.397	0.366	0.382	0.375	0.343	0.347	0.372	0.412	0.384	0.397	0.413	0.364	0.549	0.693	0.708	x	0.820	0.724	0.602	0.596	0.674	0.675	0.526	0.621	0.506
**cb3**	0.319	0.348	0.309	0.375	0.334	0.333	0.331	0.378	0.410	0.412	0.365	0.404	0.390	0.513	0.650	0.672	0.820	x	0.734	0.603	0.604	0.710	0.674	0.554	0.587	0.502
**cb4**	0.395	0.337	0.298	0.343	0.333	0.339	0.294	0.332	0.378	0.372	0.326	0.409	0.331	0.519	0.608	0.745	0.724	0.734	x	0.545	0.602	0.604	0.596	0.510	0.545	0.513
**plcd**	0.390	0.308	0.347	0.347	0.331	0.294	0.374	0.294	0.331	0.347	0.347	0.308	0.390	0.469	0.485	0.514	0.602	0.603	0.545	x	0.545	0.603	0.602	0.514	0.485	0.469
**db4**	0.331	0.409	0.326	0.372	0.378	0.332	0.294	0.339	0.333	0.343	0.298	0.337	0.395	0.513	0.545	0.510	0.596	0.604	0.602	0.545	x	0.734	0.724	0.745	0.608	0.519
**db3**	0.390	0.404	0.365	0.412	0.410	0.378	0.331	0.333	0.334	0.375	0.309	0.348	0.319	0.502	0.587	0.554	0.674	0.710	0.604	0.603	0.734	x	0.820	0.672	0.650	0.513
**db2**	0.364	0.413	0.397	0.384	0.412	0.372	0.347	0.343	0.375	0.382	0.366	0.397	0.384	0.506	0.621	0.526	0.675	0.674	0.596	0.602	0.724	0.820	x	0.708	0.693	0.549
**db1**	0.412	0.369	0.335	0.397	0.365	0.326	0.347	0.298	0.309	0.366	0.307	0.321	0.352	0.439	0.467	0.582	0.526	0.554	0.510	0.514	0.745	0.672	0.708	x	0.568	0.480
**da2**	0.372	0.395	0.369	0.413	0.404	0.409	0.308	0.337	0.348	0.397	0.321	0.403	0.385	0.592	0.771	0.467	0.621	0.587	0.545	0.485	0.608	0.650	0.693	0.568	x	0.630
**da1**	0.397	0.372	0.412	0.364	0.390	0.331	0.390	0.395	0.319	0.384	0.352	0.385	0.399	0.631	0.592	0.439	0.506	0.502	0.513	0.469	0.519	0.513	0.549	0.480	0.630	x

p-values

	aa1	aa2	ab1	ab2	ab3	ab4	plab	bb4	bb3	bb2	bb1	ba2	ba1	ca1	ca2	cb1	cb2	cb3	cb4	plcd	db4	db3	db2	db1	da2	da1
**aa1**	x	0.002	0.027	0.012	0.037	0.012	0.044	0.063	0.093	0.107	0.105	0.011	0.004	0.102	0.217	0.288	0.273	0.492	0.232	0.193	0.381	0.226	0.291	0.117	0.294	0.110
**aa2**	0.002	x	0.002	0.001	0.001	0.008	0.073	0.008	0.011	0.006	0.056	0.001	0.011	0.217	0.226	0.554	0.275	0.503	0.445	0.623	0.154	0.255	0.195	0.35	0.181	0.294
**ab1**	0.027	0.002	x	0.001	0.001	0.001	0.079	0.013	0.012	0.032	0.006	0.056	0.105	0.288	0.554	0.456	0.266	0.546	0.519	0.294	0.383	0.313	0.231	0.297	0.35	0.117
**ab2**	0.012	0.001	0.001	x	0.001	0.001	0.032	0.005	0.004	0.005	0.032	0.006	0.107	0.273	0.275	0.266	0.382	0.326	0.464	0.457	0.342	0.193	0.37	0.231	0.195	0.291
**ab3**	0.037	0.001	0.001	0.001	x	0.001	0.004	0.004	0.001	0.004	0.012	0.011	0.093	0.492	0.503	0.546	0.326	0.532	0.485	0.532	0.256	0.235	0.193	0.313	0.255	0.226
**ab4**	0.012	0.008	0.001	0.001	0.001	x	0.019	0.002	0.004	0.005	0.013	0.008	0.063	0.232	0.445	0.519	0.464	0.485	0.378	0.643	0.402	0.256	0.342	0.383	0.154	0.381
**plab**	0.044	0.073	0.079	0.032	0.004	0.019	x	0.019	0.004	0.032	0.079	0.073	0.044	0.193	0.623	0.294	0.457	0.532	0.643	0.253	0.643	0.532	0.457	0.294	0.623	0.193
**bb4**	0.063	0.008	0.013	0.005	0.004	0.002	0.019	x	0.001	0.001	0.001	0.008	0.012	0.381	0.154	0.383	0.342	0.256	0.402	0.643	0.378	0.485	0.464	0.519	0.445	0.232
**bb3**	0.093	0.011	0.012	0.004	0.001	0.004	0.004	0.001	x	0.001	0.001	0.001	0.037	0.226	0.255	0.313	0.193	0.235	0.256	0.532	0.485	0.532	0.326	0.546	0.503	0.492
**bb2**	0.107	0.006	0.032	0.005	0.004	0.005	0.032	0.001	0.001	x	0.001	0.001	0.012	0.291	0.195	0.231	0.37	0.193	0.342	0.457	0.464	0.326	0.382	0.266	0.275	0.273
**bb1**	0.105	0.056	0.006	0.032	0.012	0.013	0.079	0.001	0.001	0.001	x	0.002	0.027	0.117	0.35	0.297	0.231	0.313	0.383	0.294	0.519	0.546	0.266	0.456	0.554	0.288
**ba2**	0.011	0.001	0.056	0.006	0.011	0.008	0.073	0.008	0.001	0.001	0.002	x	0.002	0.294	0.181	0.35	0.195	0.255	0.154	0.623	0.445	0.503	0.275	0.554	0.226	0.217
**ba1**	0.004	0.011	0.105	0.107	0.093	0.063	0.044	0.012	0.037	0.012	0.027	0.002	x	0.110	0.294	0.117	0.291	0.226	0.381	0.193	0.232	0.492	0.273	0.288	0.217	0.102
**ca1**	0.102	0.217	0.288	0.273	0.492	0.232	0.193	0.381	0.226	0.291	0.117	0.294	0.110	x	0.002	0.027	0.012	0.037	0.012	0.044	0.063	0.093	0.107	0.105	0.011	0.004
**ca2**	0.217	0.226	0.554	0.275	0.503	0.445	0.623	0.154	0.255	0.195	0.35	0.181	0.294	0.002	x	0.002	0.001	0.001	0.008	0.073	0.008	0.011	0.006	0.056	0.001	0.011
**cb1**	0.288	0.554	0.456	0.266	0.546	0.519	0.294	0.383	0.313	0.231	0.297	0.35	0.117	0.027	0.002	x	0.001	0.001	0.001	0.079	0.013	0.012	0.032	0.006	0.056	0.105
**cb2**	0.273	0.275	0.266	0.382	0.326	0.464	0.457	0.342	0.193	0.37	0.231	0.195	0.291	0.012	0.001	0.001	x	0.001	0.001	0.032	0.005	0.004	0.005	0.032	0.006	0.107
**cb3**	0.492	0.503	0.546	0.326	0.532	0.485	0.532	0.256	0.235	0.193	0.313	0.255	0.226	0.037	0.001	0.001	0.001	x	0.001	0.004	0.004	0.001	0.004	0.012	0.011	0.093
**cb4**	0.232	0.445	0.519	0.464	0.485	0.378	0.643	0.402	0.256	0.342	0.383	0.154	0.381	0.012	0.008	0.001	0.001	0.001	x	0.019	0.002	0.004	0.005	0.013	0.008	0.063
**plcd**	0.193	0.623	0.294	0.457	0.532	0.643	0.253	0.643	0.532	0.457	0.294	0.623	0.193	0.044	0.073	0.079	0.032	0.004	0.019	x	0.019	0.004	0.032	0.079	0.073	0.044
**db4**	0.381	0.154	0.383	0.342	0.256	0.402	0.643	0.378	0.485	0.464	0.519	0.445	0.232	0.063	0.008	0.013	0.005	0.004	0.002	0.019	x	0.001	0.001	0.001	0.008	0.012
**db3**	0.226	0.255	0.313	0.193	0.235	0.256	0.532	0.485	0.532	0.326	0.546	0.503	0.492	0.093	0.011	0.012	0.004	0.001	0.004	0.004	0.001	x	0.001	0.001	0.001	0.037
**db2**	0.291	0.195	0.231	0.37	0.193	0.342	0.457	0.464	0.326	0.382	0.266	0.275	0.273	0.107	0.006	0.032	0.005	0.004	0.005	0.032	0.001	0.001	x	0.001	0.001	0.012
**db1**	0.117	0.35	0.297	0.231	0.313	0.383	0.294	0.519	0.546	0.266	0.456	0.554	0.288	0.105	0.056	0.006	0.032	0.012	0.013	0.079	0.001	0.001	0.001	x	0.002	0.027
**da2**	0.294	0.181	0.35	0.195	0.255	0.154	0.623	0.445	0.503	0.275	0.554	0.226	0.217	0.011	0.001	0.056	0.006	0.011	0.008	0.073	0.008	0.001	0.001	0.002	x	0.002
**da1**	0.110	0.294	0.117	0.291	0.226	0.381	0.193	0.232	0.492	0.273	0.288	0.217	0.102	0.004	0.011	0.105	0.107	0.093	0.063	0.044	0.012	0.037	0.012	0.027	0.002	x

the squared singular values of first and second pairs of singular warps as percentages of the sum of squared covariances

	**aa1**	**aa2**	**ab1**	**ab2**	**ab3**	**ab4**	**plab**	**bb4**	**bb3**	**bb2**	**bb1**	**ba2**	**ba1**	**ca1**	**ca2**	**cb1**	**cb2**	**cb3**	**cb4**	**plcd**	**db4**	**db3**	**db2**	**db1**	**da2**	**da1**
**aa1**	x	77/14	86/08	73/19	73/18	79/14	76/15	75/15	75/17	70/22	79/13	74/17	82/12	70/14	58/24	69/18	63/20	52/27	67/20	57/27	62/24	67/19	64/22	76/16	58/27	74/17
**aa2**	77/14	x	84/09	77/17	79/14	80/15	73/18	81/13	76/17	75/19	81/10	84/11	74/17	58/24	76/12	65/21	69/16	68/15	69/18	57/23	80/10	73/14	68/18	71/14	73/15	58/27
**ab1**	86/08	84/09	x	86/10	87/08	89/07	86/09	85/10	83/11	83/10	91/06	81/10	79/13	70/19	65/21	74/18	77/11	69/16	61/20	70/15	69/18	81/08	80/11	82/11	71/14	76/16
**ab2**	73/19	77/17	86/10	x	74/19	77/17	79/16	79/15	75/20	69/24	83/10	75/19	70/22	63/21	69/16	77/11	71/16	66/21	65/20	56/26	72/17	73/17	68/20	80/11	68/18	64/22
**ab3**	73/18	79/14	87/08	74/19	x	80/15	80/16	82/13	82/15	75/20	83/11	76/17	75/18	52/27	68/15	69/16	66/21	62/25	60/23	59/25	73/16	77/11	73/17	81/08	73/14	67/20
**ab4**	79/14	80/15	89/07	77/17	80/15	x	79/14	83/10	82/13	79/15	85/10	81/13	76/16	68/21	69/18	61/20	65/20	60/23	61/19	57/25	68/17	73/16	72/17	69/18	80/10	63/25
**plab**	76/15	73/18	86/09	79/16	80/16	79/14	x	79/14	80/16	79/16	86/09	73/18	76/15	58/28	57/23	70/15	56/26	59/25	57/25	64/24	57/25	59/25	56/26	70/15	57/23	58/28
**bb4**	75/15	81/13	85/10	79/15	82/13	83/10	79/14	x	80/15	77/17	89/07	80/15	79/14	62/24	80/10	69/18	72/17	73/16	68/17	57/25	61/19	60/23	65/20	61/20	69/18	67/20
**bb3**	75/17	76/17	83/11	75/20	82/15	82/13	80/16	80/15	x	74/19	87/08	79/14	73/18	67/20	73/14	81/08	73/17	77/11	73/16	59/25	60/23	62/25	66/21	69/16	68/15	52/27
**bb2**	70/22	75/19	83/10	69/24	75/20	79/15	79/16	77/17	74/19	x	86/10	77/17	73/19	64/22	68/18	80/11	68/20	73/17	72/17	56/26	65/20	66/21	71/16	77/11	69/16	63/21
**bb1**	79/13	81/10	91/06	83/10	83/11	85/10	86/09	89/07	87/08	86/10	x	84/09	86/08	76/16	71/14	82/11	80/11	81/08	69/18	70/15	61/20	69/16	77/11	74/18	65/21	70/19
**ba2**	74/17	84/11	81/10	75/19	76/17	81/13	73/18	80/15	79/14	77/17	84/09	x	77/14	58/27	73/15	71/14	68/18	73/14	80/10	57/23	69/18	68/15	69/16	65/21	76/12	58/24
**ba1**	82/12	74/17	79/13	70/22	75/18	76/16	76/15	79/14	73/18	73/19	86/08	77/14	x	74/17	58/27	76/16	64/22	67/19	62/24	57/27	67/20	52/27	63/20	69/18	58/24	70/14
**ca1**	70/14	58/24	70/19	63/21	52/27	68/21	58/28	62/24	67/20	64/22	76/16	58/27	74/17	x	77/14	86/08	73/19	73/18	79/14	76/15	75/15	75/17	70/22	79/13	74/17	82/12
**ca2**	58/24	76/12	65/21	69/16	68/15	69/18	57/23	80/10	73/14	68/18	71/14	73/15	58/27	77/14	x	84/09	77/17	79/14	80/15	73/18	81/13	76/17	75/19	81/10	84/11	74/17
**cb1**	69/18	65/21	74/18	77/11	69/16	61/20	70/15	69/18	81/08	80/11	82/11	71/14	76/16	86/08	84/09	x	86/10	87/08	89/07	86/09	85/10	83/11	83/10	91/06	81/10	79/13
**cb2**	63/20	69/16	77/11	71/16	66/21	65/20	56/26	72/17	73/17	68/20	80/11	68/18	64/22	73/19	77/17	86/10	x	74/19	77/17	79/16	79/15	75/20	69/24	83/10	75/19	70/22
**cb3**	52/27	68/15	69/16	66/21	62/25	60/23	59/25	73/16	77/11	73/17	81/08	73/14	67/19	73/18	79/14	87/08	74/19	x	80/15	80/16	82/13	82/15	75/20	83/11	76/17	75/18
**cb4**	67/20	69/18	61/20	65/20	60/23	61/19	57/25	68/17	73/16	72/17	69/18	80/10	62/24	79/14	80/15	89/07	77/17	80/15	x	79/14	83/10	82/13	79/15	85/10	81/13	76/16
**plcd**	57/27	57/23	70/15	56/26	59/25	57/25	64/24	57/25	59/25	56/26	70/15	57/23	57/27	76/15	73/18	86/09	79/16	80/16	79/14	x	79/14	80/16	79/16	86/09	73/18	76/15
**db4**	62/24	80/10	69/18	72/17	73/16	68/17	57/25	61/19	60/23	65/20	61/20	69/18	67/20	75/15	81/13	85/10	79/15	82/13	83/10	79/14	x	80/15	77/17	89/07	80/15	79/14
**db3**	67/19	73/14	81/08	73/17	77/11	73/16	59/25	60/23	62/25	66/21	69/16	68/15	52/27	75/17	76/17	83/11	75/20	82/15	82/13	80/16	80/15	x	74/19	87/08	79/14	73/18
**db2**	64/22	68/18	80/11	68/20	73/17	72/17	56/26	65/20	66/21	71/16	77/11	69/16	63/20	70/22	75/19	83/10	69/24	75/20	79/15	79/16	77/17	74/19	x	86/10	77/17	73/19
**db1**	76/16	71/14	82/11	80/11	81/08	69/18	70/15	61/20	69/16	77/11	74/18	65/21	69/18	79/13	81/10	91/06	83/10	83/11	85/10	86/09	89/07	87/08	86/10	x	84/09	86/08
**da2**	58/27	73/15	71/14	68/18	73/14	80/10	57/23	69/18	68/15	69/16	65/21	76/12	58/24	74/17	84/11	81/10	75/19	76/17	81/13	73/18	80/15	79/14	77/17	84/09	x	77/14
**da1**	74/17	58/27	76/16	64/22	67/20	63/25	58/28	67/20	52/27	63/21	70/19	58/24	70/14	82/12	74/17	79/13	70/22	75/18	76/16	76/15	79/14	73/18	73/19	86/08	77/14	x

RV coefficients

	aa1	aa2	ab1	ab2	ab3	ab4	plab	bb4	bb3	bb2	bb1	ba2	ba1	ca1	ca2	cb1	cb2	cb3	cb4	plcd	db4	db3	db2	db1	da2	da1
**aa1**	x	0.241	0.175	0.197	0.178	0.175	0.111	0.161	0.176	0.191	0.133	0.226	0.336	0.075	0.089	0.085	0.082	0.074	0.081	0.070	0.056	0.090	0.083	0.125	0.085	0.095
**aa2**	0.241	x	0.223	0.399	0.341	0.278	0.174	0.223	0.285	0.330	0.210	0.529	0.226	0.089	0.103	0.067	0.096	0.089	0.070	0.059	0.092	0.103	0.103	0.091	0.097	0.085
**ab1**	0.175	0.223	x	0.369	0.362	0.503	0.226	0.221	0.258	0.213	0.330	0.210	0.133	0.085	0.067	0.075	0.084	0.069	0.053	0.056	0.059	0.087	0.105	0.089	0.091	0.125
**ab2**	0.197	0.399	0.369	x	0.579	0.429	0.256	0.287	0.379	0.417	0.213	0.330	0.191	0.082	0.096	0.084	0.103	0.095	0.086	0.065	0.095	0.109	0.104	0.105	0.103	0.083
**ab3**	0.178	0.341	0.362	0.579	x	0.451	0.290	0.310	0.408	0.379	0.258	0.285	0.176	0.074	0.089	0.069	0.095	0.086	0.074	0.064	0.092	0.108	0.109	0.087	0.103	0.090
**ab4**	0.175	0.278	0.503	0.429	0.451	x	0.236	0.314	0.310	0.287	0.221	0.223	0.161	0.081	0.070	0.053	0.086	0.074	0.059	0.055	0.066	0.092	0.095	0.059	0.092	0.056
**plab**	0.111	0.174	0.226	0.256	0.290	0.236	x	0.236	0.290	0.256	0.226	0.174	0.111	0.070	0.059	0.056	0.065	0.064	0.055	0.057	0.055	0.064	0.065	0.056	0.059	0.070
**bb4**	0.161	0.223	0.221	0.287	0.310	0.314	0.236	x	0.451	0.429	0.503	0.278	0.175	0.056	0.092	0.059	0.095	0.092	0.066	0.055	0.059	0.074	0.086	0.053	0.070	0.081
**bb3**	0.176	0.285	0.258	0.379	0.408	0.310	0.290	0.451	x	0.579	0.362	0.341	0.178	0.090	0.103	0.087	0.109	0.108	0.092	0.064	0.074	0.086	0.095	0.069	0.089	0.074
**bb2**	0.191	0.330	0.213	0.417	0.379	0.287	0.256	0.429	0.579	x	0.369	0.399	0.197	0.083	0.103	0.105	0.104	0.109	0.095	0.065	0.086	0.095	0.103	0.084	0.096	0.082
**bb1**	0.133	0.210	0.330	0.213	0.258	0.221	0.226	0.503	0.362	0.369	x	0.223	0.175	0.125	0.091	0.089	0.105	0.087	0.059	0.056	0.053	0.069	0.084	0.075	0.067	0.085
**ba2**	0.226	0.529	0.210	0.330	0.285	0.223	0.174	0.278	0.341	0.399	0.223	x	0.241	0.085	0.097	0.091	0.103	0.103	0.092	0.059	0.070	0.089	0.096	0.067	0.103	0.089
**ba1**	0.336	0.226	0.133	0.191	0.176	0.161	0.111	0.175	0.178	0.197	0.175	0.241	x	0.095	0.085	0.125	0.083	0.090	0.056	0.070	0.081	0.074	0.082	0.085	0.089	0.075
**ca1**	0.075	0.089	0.085	0.082	0.074	0.081	0.070	0.056	0.090	0.083	0.125	0.085	0.095	x	0.241	0.175	0.197	0.178	0.175	0.111	0.161	0.176	0.191	0.133	0.226	0.336
**ca2**	0.089	0.103	0.067	0.096	0.089	0.070	0.059	0.092	0.103	0.103	0.091	0.097	0.085	0.241	x	0.223	0.399	0.341	0.278	0.174	0.223	0.285	0.330	0.210	0.529	0.226
**cb1**	0.085	0.067	0.075	0.084	0.069	0.053	0.056	0.059	0.087	0.105	0.089	0.091	0.125	0.175	0.223	x	0.369	0.362	0.503	0.226	0.221	0.258	0.213	0.330	0.210	0.133
**cb2**	0.082	0.096	0.084	0.103	0.095	0.086	0.065	0.095	0.109	0.104	0.105	0.103	0.083	0.197	0.399	0.369	x	0.579	0.429	0.256	0.287	0.379	0.417	0.213	0.330	0.191
**cb3**	0.074	0.089	0.069	0.095	0.086	0.074	0.064	0.092	0.108	0.109	0.087	0.103	0.090	0.178	0.341	0.362	0.579	x	0.451	0.290	0.310	0.408	0.379	0.258	0.285	0.176
**cb4**	0.081	0.070	0.053	0.086	0.074	0.059	0.055	0.066	0.092	0.095	0.059	0.092	0.056	0.175	0.278	0.503	0.429	0.451	x	0.236	0.314	0.310	0.287	0.221	0.223	0.161
**plcd**	0.070	0.059	0.056	0.065	0.064	0.055	0.057	0.055	0.064	0.065	0.056	0.059	0.070	0.111	0.174	0.226	0.256	0.290	0.236	x	0.236	0.290	0.256	0.226	0.174	0.111
**db4**	0.056	0.092	0.059	0.095	0.092	0.066	0.055	0.059	0.074	0.086	0.053	0.070	0.081	0.161	0.223	0.221	0.287	0.310	0.314	0.236	x	0.451	0.429	0.503	0.278	0.175
**db3**	0.090	0.103	0.087	0.109	0.108	0.092	0.064	0.074	0.086	0.095	0.069	0.089	0.074	0.176	0.285	0.258	0.379	0.408	0.310	0.290	0.451	x	0.579	0.362	0.341	0.178
**db2**	0.083	0.103	0.105	0.104	0.109	0.095	0.065	0.086	0.095	0.103	0.084	0.096	0.082	0.191	0.330	0.213	0.417	0.379	0.287	0.256	0.429	0.579	x	0.369	0.399	0.197
**db1**	0.125	0.091	0.089	0.105	0.087	0.059	0.056	0.053	0.069	0.084	0.075	0.067	0.085	0.133	0.210	0.330	0.213	0.258	0.221	0.226	0.503	0.362	0.369	x	0.223	0.175
**da2**	0.085	0.097	0.091	0.103	0.103	0.092	0.059	0.070	0.089	0.096	0.067	0.103	0.089	0.226	0.529	0.210	0.330	0.285	0.223	0.174	0.278	0.341	0.399	0.223	x	0.241
**da1**	0.095	0.085	0.125	0.083	0.090	0.056	0.070	0.081	0.074	0.082	0.085	0.089	0.075	0.336	0.226	0.133	0.191	0.176	0.161	0.111	0.175	0.178	0.197	0.175	0.241	x
